# Exploring Carceral Food Systems as Sites of Contestation and Possibility in Canadian Federal Prisons: The Food Services Modernization Initiative

**DOI:** 10.1007/s10612-022-09628-x

**Published:** 2022-04-28

**Authors:** Amanda Wilson

**Affiliations:** School of Social Innovation, Saint Paul University, Ottawa, ON Canada

## Abstract

Centering the perspectives and lived experiences of incarcerated persons, this article considers the ways food is used as a tool and site of contestation and possibility within federal prisons in Canada. Focusing specifically on the implementation of and resistance to the Food Services Modernization Initiative, I explore food as “contested terrain” within carceral systems, making visible a range of tactics of resistance employed by incarcerated persons, from testimonials and official complaints to direct collective action. In analyzing these actions and narratives, I reflect on the importance of both food justice and prisoner justice to transforming carceral food systems and call for greater acknowledgment of carceral food systems within food movement discourses and campaigns.

## Introduction

Behind bars, food holds great importance; it is a source of nourishment, a valued commodity within the informal economy, a tool of both punishment and healing and a means through which to express one’s identity (Jimenez Murguia 2018; Godderis 2006a; Graaf and Kilty 2016; Timler and Brown 2019). De Graaf and Kilty (2016: 31) note that the consumption of food within prison holds “extra-symbolic value” because incarcerated individuals are denied the opportunity to engage in many common food preparation and consumption traditions. Smith goes so far as to argue that food and its diverse meanings are “symbolic of the prison experience” (2002: 197). However, within prisons, food is also a site and tool of contestation to challenge power relations and prefigure alternative possibilities (Brisman 2008). From hunger strikes and sharing food to gardening and cooking, food can be a means to resist state violence and re-imagine post-carceral futures. Given these diverse roles and meanings, carceral food systems provide a unique and compelling area of study that implicates a range of actors and organizations both within and outside of correctional institutions.

In this article I explore food as “contested terrain” (Brisman 2008) within the Canadian federal prison system to make visible moments where food has been taken up as a tool to contest the treatment of incarcerated persons and articulate alternative possibilities. I focus on the implementation of the Food Services Modernization Initiative (FSMI), which brought in significant changes to the prison food service model and was met with both individual and collective resistance by incarcerated persons and prison advocates. The case of the FSMI illustrates how food is used, on one hand, as a weapon by the state but also as a tool of contestation by those incarcerated within the federal Canadian prison system. Drawing on publicly available testimonials of incarcerated persons, in particular articles published in the *Journal of Prisoners on Prisons*, government reports, academic literature and media articles, I interrogate the contrasting narratives of the FSMI presented by the state and by incarcerated persons. In doing so, I make visible the complex interconnections between food justice and prisoner justice, highlighting the ways the narrative about and experience of food can be used to contest current realities and enact diverse possibilities within and beyond prisons. Food and food systems can be a lens through which to imagine and enact post-carceral possibilities; however, I argue that such a task would be strengthened through greater engagement by food movements to recognize the injustices facing incarcerated persons as a “food issue.”

In exploring this topic, I am committed to centering the perspectives and lived experiences of incarcerated persons; their accounts of food in prison, how they value and relate to their food and how they see food as a tool and site of contestation. This is based on the simple assumption that incarcerated persons have a perspective worth listening to. In the language of food systems, they are consumers, producers and workers. Piché and Walby (2018: 1) contend there is great value and power in starting from the diverse standpoints of incarcerated persons:

Beginning with these standpoints forges a path toward an alternative criminology that shifts away from the dominant modes of conceptualizing and responding to acts that are criminalized and punished, which result in far too many human beings being confined to cages and subject to violent controls.

Indeed, centering the perspectives of incarcerated persons is a first step to breaking down the walls between prisons, those incarcerated and food systems to illustrate the ways carceral food systems are embedded in and connected to “outside” food systems. These interconnections mean that everyone who seeks a just and sustainable food system—not only those committed to prisoner justice—must intervene. By identifying and examining the possibilities for transformative food justice within carceral food systems in Canada, I hope to illustrate the complex interactions between food and carcerality and highlight possible points of solidarity and tension between social movements advocating for food justice (food movements) and those advocating for prisoner justice (transformative justice movements).

## Methods

While it is notoriously difficult to gain access to federal prisons for the purposes of conducting interviews with incarcerated persons, publications by incarcerated persons provide a valuable source of insight to hear directly from their perspectives. The *Journal of Prisoners on Prison* (JPP) is one such publication. The JPP is a “prisoner written, academically oriented and peer reviewed” publication that shares the perspectives of current and former incarcerated persons on prisons and the criminal justice system while also providing a broader social analysis (JPP nd). The writing and testimony of incarcerated individuals has long been seen as an important source of critical analysis on prisons (Fisher 2021; Harlow 1992; Phillips 2014). In her examination of maximum-security prisons in the US, Rhodes (2004: 7) sees great value in the testimony of incarcerated individuals, those who experience the “actual practices of confinement.” She suggests they “offer an embedded commentary that can illuminate theoretical difficulties being excavated elsewhere.” Part autobiographical testimony, part political analysis, the narratives of incarcerated individuals provide invaluable insights on the lived experience of carceral food systems. Harlow observes this duality in her discussions of the writing of incarcerated women. In reference to Ruth First’s writing, Harlow notes that “First’s narrative is more than a testimony of her personal suffering in a South African jail. Her own experience is again contextualized within a social analysis of the structure of the prison system” (1986: 519).

For this article, a search was conducted of all available archived issues of JPP between 1988 and 2019 using the search terms “food” and “meal.” Articles that did not speak to the Canadian context were excluded, as were articles that only used the terms in passing. Using these parameters, a total of 36 articles were identified, 18 of which came from a 2017 double special issue entitled “Dialogue on Canada's Federal Penitentiary System and the Need for Change.” JPP was selected for several reasons. While most publications by incarcerated persons originate from a particular institution, the JPP is one of the few that features the perspectives of incarcerated individuals across various prisons and jurisdictions. First published in 1988, JPP is also unique in that it seeks to create a space for deeper, more substantial critical reflection and discussion compared to many other publications, focusing on broader questions of penal and carceral systems as a whole.

Additional testimonials from incarcerated persons as well as other secondary data were identified through a search of Canadian news media articles and a review of relevant federal government documents and reports. Government documents included Corrections Service Canada (CSC) policy documents, annual reports from the Office of the Correctional Investigator and reports and testimony given to the Standing Senate Committee on Human Rights. For the news articles, search terms were “food service,” “food,” “meal,” “FSMI” and “prison” or “Corrections Service Canada.” A secondary search specific to an incident at Saskatchewan Penitentiary, discussed below, was also conducted, using “Saskatchewan Penitentiary,” “riot” and “food” as search terms. An analysis of the data drawn from these documents produced deeply contrasting narratives when discussing carceral food systems and the FSMI specifically.

### Unpacking Carceral Food Systems

Several scholars have noted a growing interest in connections between food and prisons (Godderis 2006a, 2006b; Sbicca 2016; Smoyer 2015, 2019; Timler and Brown 2019). These connections have been explored through several key framings: food as rehabilitation (Jiler 2009; Moore et al. 2015; Timler et al. 2019), food as a means to express one’s identity and status (Smoyer 2015; Stearns 2019; Van Hagen 2020) and food as a tool of repression and alienation (de Graaf and Kilty 2016; Earle and Phlips 2012; Gibson-Light 2018; Jones 2017; Watkins 2013; Vanhouche 2015). Building on these framings, food becomes an important site of contestation and possibility (Brisman, 2008; Godderis 2006b; Smith 2002). As Godderis (2006b: 256) notes, within prisons, food “acts as a site of contention where struggles over power, and identity (de)construction and maintenance can be played out.” Similarly, Jimenez Murguia (2018: x) asserts that “the experience of food in jails and prison can be used beyond an item of necessity for survival, but also establishing autonomy, navigating informal economies, and more importantly as an item of both control and resistance.” These acts of resistance may be individual or collective, overt or covert (Godderis 2006b: 259) and range from everyday acts of resistance (Scott 1985) such as sharing or saving food, to more formal organized actions such as hunger strikes (Brisman 2008) and formal complaints (Vanhouche 2015).

My interest is not solely in the experience and meaning of food in prison but extends to the broader food environment and the ways food circulates into and within prisons. While the experience and symbolism of food in prisons has garnered significant scholarly attention (though primarily in the US, not in Canada^1^), research is only beginning to unpack the food systems and circuits that structure those experiences and meanings in prison. Carceral studies is particularly useful in this regard as it sees prisons as part of a system and emphasizes the various ways carceral systems extend beyond the prison walls. As Wilson Gilmore (2007) explains in her seminal analysis of prison expansion in California in the 1980s, prisons shape and are shaped by the broader political economy in which they exist. In the case of California, prison expansion was spurred by surpluses of “finance capital, labour, land and state capacity” (98). Similarly, carceral food systems are influenced by the political economy of food systems^2^ as well as prevailing state ideologies on crime and punishment.

Gill et al. (2018) speak of “carceral circuits” to highlight the connections and circulations of resources, people and practices within and beyond prison walls. In doing so, they challenge the tendency to view prisons as disconnected from the rest of society. The concept of a carceral food system has a similar objective, seeking to make visible the actors, activities and relationships implicated in the provisioning of food in prisons; many of which extend within and beyond prisons—in the words of Gill et al. (2018: 184), to “give priority to the connections between, around, within and beyond carceral institutions.” As Turner (2016: 2) notes, “prisons have come to be seen not as separate, peripheral sites, but as windows onto (or even organizing principles of) modern social, political, and even economic orders.” Far from just a fixed physical barrier, the prison boundary, as defined by Turner, is composed of multiple interactions and interlinkages that connect society to spaces of incarceration. Carceral food systems are emblematic of these connections, as provisioning and consumption activities permeate the prison boundary in a variety of material and symbolic ways.

### Carceral Food Systems in Canada

There are 43 federal prisons in Canada, incarcerating 14,015 individuals at 55 institutions (Correctional Services Canada 2019a). While the vast majority of incarcerated persons find themselves in provincial or territorial prisons (24,657),^3^ those in federal custody arguably have the most intimate and prolonged experience of incarceration, with sentences ranging from two years plus a day to life.^4^ While Canada has not yet seen the same levels of mass incarceration as its southern neighbor, and the overall rate of incarceration may be declining (Statistics Canada 2021), concerns with the conditions of incarceration are just as pronounced (or, intense, numerous, prominent...). Despite the popular narrative that Canada is a positive example compared to its southern neighbor, Dawe and Goodman (2017: 130) are quick to point out that “Canada is nevertheless a quite punitive place in which ‘treatment’ and ‘rehabilitation’ operate as a sort of ‘liberal veil’ covering up a sordid postcolonial history of Indigenous, racial, and gender inequality.” There are, for instance, significant racial inequities; Indigenous and Black people are incarcerated at a much higher rate than the overall population. Indigenous people represent 4% of the Canadian population but account for 29.4% percent of the incarcerated population; and at 41.4%, the situation is even worse for Indigenous women (Office of the Correctional Investigator 2018). Black people represent 3.5% of the overall population, yet make up 8.6% of incarcerated persons (Office of the Correctional Investigator 2017). There are also concerns over chronic overcrowding and double-bunking (Ling 2019; MacAlpine 2019), inadequate health care access (Office of the Correctional Investigator 2016), cuts to prisoner pay, a lack of accountability and a flawed complaints mechanism (Standing Senate Committee on Human Rights 2019). As the Senate Standing Committee on Human Rights (2019: 8–9) recently summarized in their review of the human rights of incarcerated persons:

The stories shared by federally-sentenced persons were similar from one institution to the next and from one region to another. The committee heard that access to healthcare is inadequate, admission to gradual and structured release is insufficient, correctional programming is deficient, conditions of confinement are poor, access to remedial measures is lacking and quality and quantity of food is severely substandard.

Looking specifically at food, there are deeply contrasting narratives: the narrative perpetuated by prisons and the government as a whole, and the narrative put forth by incarcerated persons and their allies. A common view of prison food is that incarcerated persons should be happy with what they receive, perpetuating the implicit, and sometimes explicit, assumption that incarcerated individuals are in prison to be punished; that the conditions of confinement should be poor, difficult and even harmful. Take, for instance, this quote from Jeremy Laurin, spokesman for then Public Safety Minister Steven Blaney: “prisons are meant to correct criminal behaviour, not serve as a vacation home. We are confident that CSC provides meals that meet appropriate nutrition standards” (Clancy 2015). Similarly, in 2015, in response to complaints about the food at provincial prisons, Brad Wall, the premier of Saskatchewan, quipped, “if you really don't like the prison food, there's one way to avoid it and that's don't go to prison” (CBC News 2016a, b). These comments are a classic example of the “less eligibility” principle, the belief that those in prison should not experience a better standard of living than the most minimal accepted standard of living within broader society (de Graaf and Kilty 2016). Of course, such perspectives fail to acknowledge the deep injustices that permeate current criminal justice systems and the fact that incarcerated persons have rights, including the right to food. Further, they are not reflective of CSC’s stated mission, which is “encouraging and assisting offenders to become law-abiding citizens, while exercising reasonable, safe, secure and humane control” (CSC 2012).

#### Perspectives of Incarcerated Persons

The narrative presented by incarcerated persons and their advocates suggests that CSC is failing to adhere to this mission in regards to prison food. The current state of the carceral food system in Canada is perhaps best characterized as “from bad to worse.” The Office of the Correctional Investigator (2019: 3) recently referred to the food service in federal prisons as “substandard and inadequate to meet nutritional needs.” A 2018 study found that 73% of federally incarcerated persons in Canada gained weight during their incarceration. The authors of that study referred to federal prisons as “obesogenic environments” (Johnson et al. 2018).^5^ Hyper A’Hern (2017: 83), a federally incarcerated person in the Atlantic region, summed it up as, “we get fatter, while at the same time being malnourished.” Photos of meals served in prisons suggest bland, carb-heavy meals, usually featuring some sort of semi-indistinguishable stew or slop.^6^ Sean Ellacot (2017), director of the Prison Law Clinic, notes that following disciplinary court and parole, they receive the most complaints from incarcerated persons about food.

Indeed, incarcerated persons in Canada frequently cite food as a concern, critiquing the quality, nutritional value and cost. Articles by incarcerated individuals in the JPP spoke of the lack of variation as well as the lack of healthy and nutritious meals. For instance, the exclusive use of powdered milk was mentioned several times. Common characterizations in articles included “horrible,” “inadequate,” “processed pap” and “all processed.” One incarcerated person writes “we do not even know what we are eating.” The lack of accommodation of special diets (for religious or medical reasons) was also raised several times, as was the belief that food was used as a tool of discipline. However, in addition to the many critiques of the food in prison, incarcerated persons also spoke of actions to improve their food and food as a way to build community and engage in collective action.

Gayle Horii (1999: 9), quoting her friend Jo-Ann Mayhew, a fellow incarcerated person who had recently passed, spoke of a victory in December 1989 at the Prison for Women in Kingston: “as of Dec. 1, we are allowed to take one serving of salad ex. tomatoes or cereal or dessert ex. fruit from the dining room!” Gregory McMaster (1999: 94) describes the food in solitary confinement in the late 1990s, highlighting the small but meaningful opportunities to engage in collective action:

Meals can be a test of your character and resolve. The food is often undercooked, always cold, and the portions small. Your so-called brothers in the kitchen have forgotten all about you. Out of sight, out of mind. Prison food was never anything to write home about, but a trip to the chow hall is like a four-star restaurant compared to this. You close your eyes and eat the slop. It is the only sustenance you are going to get. Once in a while untouched trays are fired from all the cells in protest. *The show of unity does little to bolster your hunger pains, but it serves to bolster your pride* (emphasis added).

These vignettes from Gayle Horii and Gregory McMaster are powerful examples of food as everyday acts of resistance, actions that are relatively safe and require little coordination or organization, but offer important material gains that draw on a shared culture of resistance (Scott 1989). Jarrod G. Shook (2015: 75) describes how, as a kitchen worker, he prepared Omar Khadr’s^7^ meals while they were both incarcerated at Milhaven: “though he did not know it, I always took care to make sure that his tray was as full as possible. I often found myself defending him against the bigoted personal attacks of ignorant food service workers and prisoners alike.” Here Shook highlights the intimacy of preparing food for someone else, almost as a form of solidarity between incarcerated persons, even if it is unknown to both parties.

Kim Pate (2003), now a Senator, recalled the time she first met Gayle Horii, the incarcerated person referenced above, in British Columbia. She described how Gayle hosted her and prepared them a meal: “Gayle not only nourished my mind, she also fed my body in her ‘cage’ that late January day and early evening. She had retained eggs, lettuce, and other salad ingredients that she offered me in the form of a delicious and incredibly nutritious meal” (164). This small story highlights the role that food plays in providing a sense of autonomy and humanity for incarcerated persons and how food is a means to enact an ethics of care and solidarity, something also noted by de Graaf and Kilty (2016) in their analysis of women’s experience of food within prison.

These voices highlight the nuances and complexities in how incarcerated persons experience and relate to carceral food systems. It also emphasizes that they are active participants in these systems; analyzing, reflecting and taking action. While government narratives present prison food as meeting nutritional requirements and therefore “good enough,” incarcerated individuals present a more complex narrative, positioning carceral food systems as sites of oppression and harm, but also as sites of solidarity, resistance and care.

### Food as a Site and Tool of Contestation: The Food Services Modernization Initiative

In 2014, the federal government instituted a suite of changes to federal prison food services as part of their Food Services Modernization Initiative (FSMI). The FSMI introduced a new “National Menu” for all federal prisons and shifted food preparation from a decentralized in-house model to a centralized “cook-chill” system whereby meals are prepared at five regional hubs, packaged and then chilled prior to distribution; individual prisons then reheat the meals. The National Menu is said to adhere to the Canada Food Guide, providing 2,600 cal per day, the recommended daily intake for “low-active” males between 19 and 50. Previously, food service was decentralized: Menus were developed at a regional or individual institutional level, with prisons preparing meals in-house with internally managed inventories (CSC 2016, 2019c). The types of foods served also shifted; fresh milk was replaced with powdered milk, the selection of vegetables and meats were reduced and more expensive grains were removed (Office of the Correctional Investigator 2017). According to the colorful infographic produced by CSC (see Appendix A), the FSMI modernizes food services and is “efficient, cost effective and ensures consistency in food preparation across the country” (CSC 2016). They claim the rationale for the centralized food production is to “maximize resources and minimize waste.” (CSC 2019b).

Under the current model, Correctional Services Canada spends a maximum of $5.41 per day for food per individual, a slight increase from the $4.98 when the FSMI was first introduced (Office of Correctional Investigator 2018). Hospitals and other public institutional settings have experienced similar shifts toward centralization and ‘cook-chill’ technology; however, they have maintained higher per serving food allocations. For comparison, in 2012, Ontario hospitals spent between $7–8 per day on food per patient (Anderson and Um 2017), while estimates for 2016 were between $8–15 per day (Luz Mejia 2016).^8^ Jones (2017) notes two additional crucial differences between food in prisons and other institutional settings: within prisons, food is used as a tool of behavior modification and as a form of punishment.

The FSMI also led to a reduction in the number of employment and training opportunities for incarcerated persons as there was no longer a full kitchen at each institution preparing meals (The Office of the Correctional Investigator 2015). It led to the cancelation of the Culinary Arts Program in at least one prison, a certification that enabled incarcerated persons to get a job in the food service industry once on the outside. According to Trevor D. Bell (2017: 210), incarcerated at Mission Institution in BC, this program “was one of the longest running and successful programs at this facility, and was truly revered as extremely beneficial by all staff and prisoners.”

Rather than an isolated policy decision, this change was a product of then Prime Minister Stephan Harper’s “tough on crime” policies that fed into the Conservative’s Deficit Reduction Action Plan (DRAP), a plan to achieve a balanced budget through a $4 billion reduction in government spending (Comack et al. 2015; Piche 2012). Beyond efforts to cut costs, the FSMI should be understood within the broader processes of “roll-out” of neoliberalism (Peck and Theodore 2012). Privatizing the federal prison food service might seem the more obvious choice, in line with the contracting-out and “unbundling” of public institutions.^9^ However, the decision to implement the FSMI is an example of the shift in state function and institutional restructuring—toward a more “punitive social policy regime” (Peck 2003: 225) where incarcerated individuals are forced to contribute to the costs of their incarceration. The centralization and standardization of food service makes it easier for the state to appear to be accountable to their mandate, all the while increasing their capacity to control and discipline incarcerated populations.

#### Perspectives of Incarcerated Persons

According to CSC, the FSMI was a glowing success: “This initiative has created a leaner more efficient process for planning, preparing, and serving over 30,000 meals each day with a cost savings of over six million dollars” (CSC 2016). However, incarcerated persons present a very different picture. These changes had, and continue to have, significant consequences for the quality and quantity of food as well as the ability of incarcerated persons to enact choice over their foods and exercise their right to dietary accommodations for religious or medical reasons. A 2017 special issue of the JPP brought together the voices of federally incarcerated persons across the country speaking out against the changes brought about by the Conservative government. These articles, along with testimonials of incarcerated persons identified in media reports since the introduction of the FSMI, are unanimous in framing the changes to the food services as disastrous.

Individuals incarcerated at Mission Institution put food at the top of their list of concerns in reference to changes brought in by the Conservative government: “After many discussions amongst the population here, not surprisingly, we all agreed that food is the highest priority on the top ten list” (Chow 2017: 232). Jean-Paul Aubee, incarcerated in British Columbia, asserts that “the food is causing people to experience diarrhea, nausea, vomiting. I have experienced this myself many times” (Clancy 2015). Concurring with Aubee, an anonymous group of incarcerated persons at Kent Institution in British Columbia wrote that “approximately 20% of the penitentiary population here suffers severe digestive problems due to the food forced upon us,” which has led to “bloody anal discharge, bloody stool, lower intestinal cramping and bloating, constipation and diarrhea, as well as stomach pains” (as quoted in Shook and McInnis 2017: 286).

Hyper A’Hern (2017: 83) paints a similar picture of the food quality during his time in prison in Atlantic Canada:

On paper, we are eating chicken cacciatore and meat pie. In reality, we are eating shards of processed chicken in a sauce that could not be described as revolting due to the glaring inaccuracies of this statement and a pie that is 80 percent fake potatoes and some spaghetti sauce.

Here one can see how CSC can use things like the National Menu to create a particular public discourse that may in fact be far from reality. A’Hern also noted finding bits of plastic, metal and bugs in the food. Speaking to the low quality of food following the shift to the centralized cook-chill method, Ronald Small (2017: 239), incarcerated at Mission Institution in British Columbia, says he “witnessed the kitchen staff hanging their heads in shame because of what they are forced to serve us.” He also observed a high level of food waste, simply because incarcerated persons would refuse to eat the food they were served. Food waste was a theme raised by several incarcerated individuals; ironic given that one of the key rationales for instituting the FSMI was to reduce food waste.

A group of anonymous incarcerated persons at Kent Institution in British Columbia also make mention of the declining quality of food, noting there used to be a healthier selection of foods when meals were cooked on-site:

The diet that is forced upon us consist of items that are classified as scoop-ables, that is they are served out as slop. All of the meals are smothered in sauces that give no nutritional value, and are loaded with artificial thickeners colors and preservatives. The food appearance is grotesque, consistent with vomit. The taste is often worse than the appearance. (Anonymous Prisoners Kent Institution 2017: 266).

They also note that the changes have taken away their ability to have input into what foods they are served and how it is prepared. An anonymous incarcerated person at Bath Institution, Ontario, connects the loss of healthier foods to the loss of employment opportunities both inside and outside prison:

The food served on the line is no longer edible due to the new procedures such as freezing. Due to the change in food services I must now purchase extra food at the canteen, which is far from ideal as these items are not healthy. I would like to see that the government make kitchen work a job training program. This would assist with employment outside of the prison that would teach marketable skills, while providing nutritious food to prisoners. (Anonymous Bath Prisoner # 1 2017: 114).

Trevor Bell (2017: 209), incarcerated in British Columbia, states bluntly, “The current status of the dietary food delivery program within the Pacific Region is a monumental waste of taxpayers’ money,” calling the switch to the cook-chill system “nothing more than a punitive action” to “inflict pain upon the penitentiary population.” They note the switch significantly decreased the quality of the food, leading to “serious health concerns” among incarcerated persons, and that “the vast majority of the meals are not in any way edible.”

Rachel Fayter and Sherry Payne (2017: 19), incarcerated at Grand Valley Institution, Ontario, assert that the introduction of the National Menu decreased their access to healthy foods, arguing that “healthy menu items were removed and replaced with canned goods and processed, unhealthy food.” They note the changes have had broader implications beyond nutritional value:

By standardizing what we eat for every penitentiary across the country, we have less variety and choice, resulting in less cultural expression from each region of the country and cultural groups within each region. Many women enjoy cooking and baking, which is a positive, pro-social activity. Restricting our choices makes us feel worthless and inferior. The unhealthy food negatively affects our physical health. (19).

Here, Fayter and Payne highlight the diverse roles that food plays within prison and how CSC’s policy change ignored the important role that food and food preparation play in peoples’ lives, to the detriment of incarcerated persons.

A group of anonymous incarcerated persons at Fraser Valley Institution for Women, British Columbia, remarked that one of the small but impactful changes was a reduction in the use of the barbecue from once every two weeks to once every month. One individual also mentioned that all food items brought in (by visitors, for instance) must be “pre-packaged and store bought” (Anonymous Prisoner #4 Fraser Valley Institution for Women 2017: 49). It is hard to see these changes as anything but punitive; aside from the marginal savings on propane, there is not much of a “cost-savings” rationale here. One can easily imagine how homemade goods would have a particular significance for incarcerated persons; again, illustrating how changes to the carceral food system can be a tool to dehumanize and weaken social bonds. Similarly, an incarcerated individual at Bath Institution, Ontario, lamented the impact of losing “food nights,” which, because they presented an opportunity to cook and socialize together and often involved family and community members, were an important source of community building with those outside of prison (Joseph 2017: 123). As racialized communities are vastly overrepresented within prison populations, the ability of incarcerated persons to access cultural foods, through homemade goods and food nights, takes on added significance.

The Inmate Committee^10^ at Bath Institution conducted a consultation with incarcerated persons to generate a list of things they wanted changed. At the top of their list was the reinstatement of group food drives. They were upset that they were no longer able to organize food drives to raise money for organizations in the community they cared about—something that enabled them to maintain a connection with the community:

Group food drives enabled us to maintain community contact and raise money for organizations such as the Make a Wish Foundation. I would like to see a return to the previous policy and for CSC to allow groups to raise money through pizza and chicken sales, cultural food drives, and the like. (Joseph 2017: 114)

The fact that this was the first issue raised speaks to the important social role that food plays in allowing incarcerated persons to enact agency and contribute to the broader community.

Some prisons use a food service model called “Small Group Meal Preparation” (SGMP), where incarcerated persons prepare their own food using groceries purchased from a central grocery list. According to a CSC document from 2013, of the 55 federal prisons, 28 used the “Central Feeding” food services model, while 25, typically minimum or medium security facilities, used SGMP. However, incarcerated persons have reported that institutions that previously used SGMP have now switched to Central Feeding (Chow 2017). Even in those prisons that have maintained the SGMP model, incarcerated persons have voiced concerns. The weekly stipend they receive to purchase items has stagnated at $35/week for years while prices have gone up and the variety of foods has greatly decreased (Fayter and Payne 2017). The SGMP model was seen to give incarcerated individuals greater control and autonomy and also helped them build skills in food preparation and budgeting: “Prisoners who have never prepared a meal in their lives have become quite proficient at it and this program instils in them a variety of skills that they can take with them when they re-enter the community” (Anonymous prisoner #12 Beaver Creek 2017: 155).

The Inmate Committee at Joliette Institution in Quebec, which uses the SGMP model, noted that food items that were previously available to purchase as part of their weekly meal allowance are no longer available or are now only available through the canteen (which they must purchase separately with their own money). They also found it increasingly difficult to access fresh fruits and vegetables (Établissement Joliette pour femmes 2017). De Graaf and Kilty (2016) note concerns over the high cost of food in their research with federally incarcerated women, a concern echoed by incarcerated women at Fraser Valley Institution, who argued the weekly rate should be increased to $50 to keep up with inflation and cost increases. They also wanted to see the return of healthier items that were removed from the grocery list, such as “mixed nuts, popcorn for snacks, almond milk without the corn syrup” (Anonymous Prisoner #4 Fraser Valley 2017: 52). Here one can see a familiar pattern; the FSMI has reduced the choice and autonomy of incarcerated persons, all while forcing them to consume less healthy food options. Standardization and centralization make it more difficult (though not impossible) for incarcerated individuals to enact agency over their food. The narrative put forth by CSC seeks to mask these detrimental impacts under the guise of efficiency and consistency, but the reality is that narrowing the choices incarcerated persons can make, whether it be organizing a food drive, preparing their own meals or purchasing healthier grocery items, amounts to punitive and harmful social policy.

#### Ripple Effects

As the Joliette Inmate Committee noted, the poor quality and quantity of food served has forced many incarcerated persons to supplement their diet with food purchased, out of pocket, at the canteen. However, this was made more difficult by room and board deductions introduced in 2013. Incarcerated persons who work now have between 22 and 30% of their daily wage for food, accommodations and telephone^11^ deducted from their pay (Fayter and Payne 2017; Shook 2015). The maximum daily wage is $6.90; however, only a small minority earn this. In reality, the average wage for an incarcerated worker is closer to $3 a day. This means that prisoners have even less money to supplement their diet through the canteen. An incarcerated individual from Bath Institution makes this connection explicit: “This policy change has made the purchase of food and vitamins unaffordable so one cannot compensate for the cuts to food quantity and quality” (Joseph 2017: 117). In addition to this, incentive pay was also eliminated.^12^ The elimination of the incentive was part of several changes introduced under an “Offender Accountability” initiative. While CSC estimates that the elimination of incentive pay has led to an annual savings of $1.747 million (CSC 2015), the name attached to the policy change suggests cost was not the primary motivation. This example highlights the importance of seeing prison food as part of the broader carceral food system so that the connections between different policy changes are made visible and the ways they mutually reinforce one another are better understood. The negative impacts of the FSMI were compounded by the Offender Accountability initiative, curtailing the purchasing power of incarcerated individuals at a time when they were forced to rely on it even more.

These testimonials of incarcerated persons are supported by the conclusions of the Senate Standing Committee on Human Rights and the Office of the Correctional Investigator. In the Senate’s (2019: 23) report on the human rights of federal prisoners, they note that:

In every penitentiary the committee visited where individuals did not cook their own food on site, senators were informed that the food is of poor quality and is often served cold or overcooked. Senators also heard that portion sizes are inadequate and do not meet the needs of fully-grown adults.

Similarly, the Office of the Correctional Investigator regularly raises questions about the prison food services in their annual reports (see 2014–2019). In 2015, the Correctional Investigator suggested that the provision of healthy and nutritional food does not appear to be a priority within the FSMI and questioned whether it was even possible to provide healthy food given the low per diem allocated. They connected the shift to cook-chill production to the “ever tightening security regime of limited and controlled inmate movements” (33). From this perspective, the FSMI is not about instituting a “more efficient process” for service provisioning, rather it is an attempt to use food as a tool of control and punishment.

#### Formal Complaints: Food-Related Grievances

**Fig. 1:**
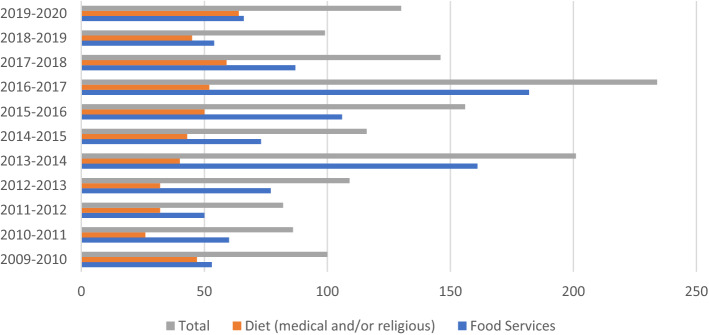
Complaints, as recorded by the Office of Correctional Investigator 2009–2020. *In certain years (2015–16, 2016–17) complaints related to Food Services were divided between two different categories—one specific to Food Services, and another, a subsection on Food Services under Conditions of Confinement. In years where both categories appeared, the total number is included from both

In addition to publishing articles and testimonials, incarcerated persons have also submitted formal complaints about the prison food service. Figure 1 tracks the number of grievances related to food as recorded by the Office of the Correctional Investigator between 2009 and 2018. One can observe an overall increase in complaints since the implementation of the FSMI. Food services, as a point of discussion, did not feature prominently in the Office of Correctional Investigators annual reports before 2014–2015 when the FSMI was instituted. In fact, the Office of Correctional Investigator noted in 2019 that “in 2015–16, inmate contacts and complaints to this Office related to meal quality spiked” (2019: 57). While the overall majority of complaints in prison are for things other than food services,^13^ these numbers highlight that food is a consistent and increasing concern of incarcerated persons.

#### Collective Action at Saskatchewan Penitentiary

As the previous section demonstrates, one response incarcerated persons have taken is to lodge formal complaints using the (admittedly imperfect) grievance systems made available to them. A second response has been writing articles and providing testimonials in media. A third response has been forms of direct, collective action. Perhaps the most significant instance of collective action in response to the changes to food services occurred in December 2016 at the Saskatchewan Penitentiary, which saw an escalating conflict between incarcerated persons and staff over food and working conditions for incarcerated persons working in the kitchen. This incident has been typically referred to as a “riot,” however this term downplays the political and organized nature of the actions. This was not a spontaneous outburst, but rather an escalation of collective action articulating a claim and seeking to address an issue. According to the Office of Correctional Investigator, 131 of the 475 incarcerated persons in Saskatchewan Penitentiary at the time participated in the action, a participation rate of 28%. The majority of incarcerated men at this prison are young and Indigenous (Office of Correctional Investigator 2017), not surprising when you consider that Saskatchewan has the highest proportion of Indigenous incarcerated persons in Canada (78%), outside of Nunavut and the Northwest Territories (Department of Justice 2017).

According to the Office of Correctional Investigator (2018) the conflict began when incarcerated persons delivered a detailed list of concerns related to food services and working conditions to management. This grew into a walk-out and strike by incarcerated persons working in the kitchen as they protested the small portions of scrambled eggs they were meant to serve. Other incarcerated persons then began refusing to work or attend school and/or programs in solidarity with the striking kitchen workers. When several meetings and discussions with prison management failed to resolve the issue to the satisfaction of those incarcerated, tensions escalated and, on the afternoon of December 14, physical altercations, large-scale disturbances and destruction of property left two incarcerated individuals with serious injuries and one dead. Prison guards used more than 36 kg of pepper spray as well as shotgun pellets to put an end to the “riot.”

In its report on the incident, the Office of Correctional Investigator concluded that the collective action was directly related to dissatisfaction with the food service. In interviews with incarcerated persons, investigators identified four categories of concerns: food shortages, particularly shortages of protein servings; the selection, replacement and substitution of food items; the quality and quantity of food served; and the working conditions of kitchen workers, including their treatment by CSC kitchen staff (2018: 44). Amelia Bloomfield, the mother of an incarcerated individual who worked in the kitchen, recounts that her son told her about the food issues that precipitated the riot: “He said they’re not getting enough food … A lot of people are hungry, not just him” (CTV News 2016a, b). Devon Paskimin, incarcerated at Saskatchewan Pen, explained to a local newspaper that the quality and quantity of food was the latest in a series of issues: “It’s kind of like the last straw, the quality of the food, the quantity, the portion sizes—they were small and the dishes from the kitchen weren’t good” (White-Crummey 2016). Even the union representative for the correctional officers notes portion sizes as a cause of the riot: “In this situation there [were] some issues with the kitchen workers, and the kitchen workers being inmates were refusing to go to work and were having some discussions over portion sizes” (650 CKOM Saskatoon 2016).

Yet the National Board of Investigation, an internal investigative body, initially concluded that the riot was a spontaneous and random event, unrelated to food service issues, and that it could not have been predicted (CSC 2018). Given the accounts of incarcerated persons, among others, it is difficult to understand how CSC came to that conclusion. It seems impossible that they would not have come across these issues in their investigation; perhaps what seemed unconceivable was that incarcerated persons had legitimate grievances and would take such strong, organized collective action to address them.

Far from just a “spontaneous riot” the actions at Saskatchewan Penitentiary were rooted in solidarity, resistance and collective action. The efforts and commitment of these incarcerated persons, predominantly young Indigenous men, is inspiring. That they were met with violence and brute force, and that the internal report fails to acknowledge the reasons behind their actions, only underscores the importance of making visible the ways incarcerated persons engage in collective action and resistance within carceral food systems. It is also telling that in a follow-up visit to Saskatchewan Penitentiary, investigators from the Office of the Correctional Investigator (2017: 35) found there were still “continuing issues with food at this facility.”

#### The FSMI Marches On …

Despite the many grievances and push back from incarcerated persons and their advocates, the FSMI remains in place. In April of 2019, CSC did complete an internal audit of Food Services. The report flagged several problems that incarcerated persons have been raising for years. For instance, it found that the National Menu failed to meet the Canada Food Guide guidelines on 6 of the 28 days of the menu cycle.^14^ It also found the information management system for special diets was not being used consistently, increasing the likelihood that special diet requirements were not being followed. Further, the Quality Assurance Program was not consistently implemented, increasing the likelihood of food contamination, and staff had not been fully trained according to National Training Standards to ensure food was being prepared in a “healthy and safe manner” (CSC 2019c: 5). Concerns were also raised about inadequate portion sizes and the disconnect between the maximum cost per meal allocated and actual production costs, meaning there is a lack of evidence that CSC is able to produce a healthy and nutritious meal at the allotted per diem. In response, CSC says it has developed a Management Action Plan—but it is unclear what this consists of and what, if any, effect it will have. What does seem clear is that FSMI is not about instituting a “more efficient process” for food provisioning, rather it is an attempt to use food as a tool of control and punishment of incarcerated persons.

This discussion is not intended to suggest that prior to 2014 food in federal prisons was of a high quality; far from it. However, the changes made as part of the FSMI further entrenched and exacerbated existing problems. Given the important role that food plays in the lives of incarcerated persons, the impacts of the FSMI have been far reaching. It is not just the decrease in quality and quantity of food that is significant. As an anonymous incarcerated individual at Beaver Creek Institution in Ontario notes:

The quality and quantity of food has always been an issue in penitentiaries, which has been further exacerbated with the introduction of a central food preparation centre … Previously, each institution had its own kitchen where staff and prisoners worked together. The prisoners learned valuable skills that could easily be transferred to the community through the example set out by staff. They learned alternative ways of proper comportment. (Anonymous Prisoner #12 Beaver Creek 2017: 155).

The centralization of food production represents a loss of autonomy on the part of incarcerated persons. The new system has made it more difficult for incarcerated persons to address grievances about their food, to secure a special diet, to participate in paid employment in the prison kitchens and to build community and meaningful relationship both inside and beyond the prison walls.

### Food as a Site of Post-Carceral Possibilities?

This article presents an example of incarcerated individuals working to disrupt processes of neoliberal institutional restructuring and the narratives surrounding it. Rather than a stable “fait accompli,” the FSMI highlights what Peck (2003: 230) refers to as the “the contingent and uneven outcomes associated with the ‘departicularized commonplaces’ of postwelfarist policy-making and penal statebuilding.” The changes implemented as part of the FSMI have had, and continue to have, detrimental impacts on the lives of incarcerated persons. Incarcerated persons have responded by sharing their grievances, lodging formal complaints and, in at least one case, engaging in direct collective action. While the resistance to food services changes was largely internal, informal and individual, it was nonetheless significant. In the stories, actions and testimonials offered by incarcerated persons it is clear that food holds a range of meanings behind bars—as a tool of community building and solidarity, an assertion of autonomy and a site of resistance.

This case highlights the utility of contextualizing prison food within carceral food systems to fully understand how a change to food service shapes and is shaped by other shifts in penal policy. It also highlights the importance of building bridges and relationships between incarcerated persons and allies on the outside so the efforts of incarcerated persons do not go unnoticed and unsupported. Incarcerated persons and their allies cannot rely on existing institutional mechanisms to create the change that is so badly needed. Even with a Correctional Investigator that is supportive of many of their concerns, there are few signs of change or accountability, despite multiple complaints logged by incarcerated persons and their allies, not to mention critical media reports.

As carceral food systems implicate not only the prison-industrial complex but the broader food system, the food movement could be a powerful ally in efforts to change them. Yet struggles over carceral food systems have rarely, if ever, been interpreted as a “food movement issue.” For instance, a review of Food Secure Canada’s website and blog archive conducted in the spring of 2020 found only six references to the word “prison.” For comparison, there are 262 references to “organic” and 414 references to “school food.” Similarly, a search of Food Secure Canada’s Twitter feed found only three references to “prison” since they joined Twitter in 2017. While Food Secure Canada is not the only organization that could be seen as representing the food movement, as the only pan-Canadian organization with the explicit mandate to build an alliance of organizations and individuals advocating for more healthy, just and sustainable food systems, it is widely seen as the voice or leader of the food movement in Canada. A similar trend can be observed in research by food movement scholars in Canada and beyond. Multiple academic searches for literature speaking to both prisons and the food movement generate scarce results—and the vast majority are from a single author in the US, Sbicca (2012, 2015, 2019).^15^

In Sbicca’s research (2016: 1359) examining instances of food justice grounded in the realities of incarcerated geographies in the US, he suggests that connecting food justice with restorative justice provides a “unique set of strategies to stanch the flow of people into prison.” He uses the concept of restorative food justice to highlight the transformative possibilities that emerge from this pairing. Building on Sbicca, I prefer to envisage transformative food justice as a means to explore the ways food systems and prisons are connected and how those connections offer possibilities for those seeking to fundamentally re-imagine food systems and forms of punishment. Transformative justice takes a slightly more expansive and radical perspective on justice, seeking not only to restore relationships between those who have harmed and been harmed, but to transform the structural inequalities that create the social conditions in which harm occurs and imagine an entirely different form of accountability (Kelly 2011; Kim 2018; Mingus 2019; Palacios 2016). From this perspective, an examination of carceral food systems can serve to counteract the dominant belief, as articulated by Davis (2003: 9), that prison abolition is both “unthinkable and implausible.” As noted at the outset of this paper, food is a powerful force within prisons, and it could be a powerful force in their transformation. As a tool of self-expression, relationship building and solidarity, food and food provisioning invite us to see the humanity and shared struggle within others and to imagine possibilities beyond current carceral realities.

## Conclusion

As this article makes clear, incarcerated persons are definitely talking about food and their food system. Yet it is equally apparent that the food movement is not yet talking enough about incarcerated persons and prisons. Discussions of food in institutional settings remains largely focused on hospitals and schools, yet the stories from incarcerated persons shared here clearly highlight severe shortcomings in the federal government’s approach to prison food services. Food has long been understood as an important tool and site of social change, a lens through which to challenge relations of power and build new relationships, structures and systems. However, this lens has yet to be extended to the carceral system in any meaningful way. Building bridges between food justice advocates and prisoner justice advocates could help to articulate a vision of transformative food justice grounded in post-carceral futures.

Just as Minkoff-Zern (2017) and others argue that labor and workers’ rights must be given more attention within movements and campaigns for sustainable food systems, I argue that incarcerated persons and carceral food systems must be included in our conceptualizing of food systems and food justice. Food movements have been criticized for their focus on “good food” politics and environmental and health concerns related to food, as well as for highlighting alternatives rather than directly confronting sources of oppression and exploitation within the dominant food system (Alkon and Guthman 2017; Holt-Gimenez 2017; Levkoe and Wilson 2019; Myers and Sbicca 2015; Sbicca 2018). Recognizing the injustices occurring within carceral food systems as a food movement issue and part of the struggle to transform our food systems represents one way for the food movement to engage in a deeper acknowledgment of the structural inequalities in the food system and build solidarity with other social movements. It offers a possible opening to build stronger links between food justice and transformative justice and encourage more critical and transboundary analysis among food movement actors. Resistance to the FSMI by incarcerated persons is but one example of how carceral food systems highlight linkages and potential points of solidarity and, in doing so, open up the possibility to build an integrated vision and practice of transformative food justice.

## Appendix

Appendix A: “Feeding Offenders at CSC” CSC 2016

## Footnotes

1 A review of the literature found that, out of 51 articles, only nine spoke to food and foodways in Canadian prisons.

2 See, for instance, Struthers (2019) for an excellent historical analysis of prison farms as manifestations of state colonial power.

3 These figures decreased considerably in response to the COVID-19 pandemic as both provincial and federal institutions were pressured to reduce the number of individuals in custody for health and safety (Malakieh 2020; Prison Pandemic Partnership 2021). It remains to be seen whether this decrease will be sustained over the long run or whether it is merely a temporary dip, thus I have used the pre-COVID figures in this paper.

4 For those unfamiliar with the Canadian penal context, some additional details may prove useful. The majority of incarcerated individuals find themselves in provincial prisons in part because of the difference in length of sentences, but also because those awaiting trial are held in provincial facilities. Individuals “in remand,” awaiting trial, far outnumber incarcerated individuals that have been sentenced (Malakieh 2020). In Canada, those imprisoned under a life sentence are eligible for parole after having served 25 years, in the case of first-degree murder convictions, and between 10 and 25 years, for second-degree murder convictions.

5 While I consider obesity to be a problematic term often used to shame and vilify bodies of different shapes and sizes, I felt the terminology was acceptable here as it illustrates the role that the prison environment plays in undesired weight gain.

6 See, for instance, the Office of the Office of the Correctional Investigator 2016–2017 Annual Report https://www.oci-bec.gc.ca/cnt/rpt/annrpt/annrpt20162017-eng.aspx

7 Omar Kadhr is a Canadian citizen who, at the age of 15, was held as a prisoner at Guantanamo Bay for ten years in response to his alleged participation in attacks against US soldiers in Afghanistan. In 2012 he was repatriated to Canada and served an additional three years behind bars. In 2017 the Canadian government paid him a $10.5 million settlement for violation of his Charter rights.

8 Interestingly, recently there has been a push to reject this standardized and technological approach to food preparation within some schools and hospitals. See, for instance, Nourish (https://www.nourishhealthcare.ca/), a multi-year collaborative project to transform food in health care.

9 Indeed, many provincial prisons have also gone a step further to fully contract-out the food services. For instance, in Ontario, Compass Group, one of the largest if not the largest food service company in the world, holds the contact for provincial institutions, providing meals using similar ‘cook-chill’ processes.

10 While inmate is not a term I would normally use, this is the official title of the group, thus to avoid confusion I make use of it here.

11 In addition to this, prisoners are still responsible for the cost of individual phone calls.

12 Incentive pay was an additional amount paid to incarcerated individuals working in CORAN industries (manufacturing, textiles, construction and prison services).

13 For instance, the total number of complaints logged for 2018–2019 was 5,291.

14 Canada’s Food Guide underwent a significant overhaul in 2019; it is even less likely that the National Menu is consistently meeting the new updated guidelines. See https://food-guide.canada.ca/en/ for further details.

15 Search terms used: “food movement” or “food activism” + “prison” or “jail” or “carceral.”
